# Antiulcer effects of *Zataria multiflora* Boiss. on indomethacin-induced gastric ulcer in rats

**Published:** 2018

**Authors:** Mohsen Minaiyan, Sayed-Ebrahim Sajjadi, Kamran Amini

**Affiliations:** 1 * Department of Pharmacology and Pharmaceutical Sciences Research Center, School of Pharmacy and Pharmaceutical Sciences, Isfahan University of Medical Sciences, Isfahan, Iran*; 2 * Department of Pharmacognosy, School of Pharmacy and Pharmaceutical Sciences, Isfahan University of Medical Sciences, Isfahan, Iran*; 3 * Schools of Pharmacy and Pharmaceutical Sciences, Isfahan University of Medical Sciences, Isfahan, Iran *

**Keywords:** Zataria multiflora, Gastric ulcer, Gastric acid, Pepsin, Ulcer index

## Abstract

**Objective:**

*Zataria multiflora* has been reported to have several medicinal properties including antioxidant, antibacterial, antispasmodic, and expectorant activities. This study aimed to investigate the effect of *Z. multiflora* hydro-alcoholic extract (ZMHE) on peptic ulcer caused by indomethacin in rats.

**Materials and Methods:**

ZMHE was prepared by maceration, condensed by rotary evaporator and dried by a freeze-drier. In this study, 72 male Wistar rats were randomly divided into 12 groups, six in each including: normal rats, control rats, ranitidine-treated, and animals that were treated with ZMHE (100, 200, 400 mg/kg). Parenteral and oral treatments were done 1 and 2 hours, respectively before gastric ulcer induction by indomethacin (25 mg/kg, i.p.). After 6 hours, animals were sacrificed, two sides of the stomach were ligated and gastric contents and tissues were investigated in terms of pH and biochemical and histologic parameters, respectively.

**Results:**

ZMHE (at all doses) considerably decreased the ulcer area and ulcer severity in comparison to control group after oral and parenteral administration. Oral administration of this extract increased the pH of stomach contents while diminished pepsin activity in a dose-dependent manner. Following parenteral treatment, a significant difference in pH of stomach content was observed only by ZMHE 400mg/kg in comparison to control group. The myeloperoxidase (MPO) enzyme activity decreased in groups that received the extract via both oral and parenteral routes.

**Conclusion:**

It might be concluded that ZMHE could protect against experimental gastric ulcer induced by indomethacin and this action is probably mediated via reducing acid secretion and pepsin activity besides enhancing tissue antioxidant capacity.

## Introduction

Peptic ulcer is one of the most important and common disorders of the alimentary system occurring due to damages in mucus and sub- mucosal layers of the alimentary tract (Raghuveer and Chakarvarthy, 2013 ▶). This disease results from imbalance between defensive factors including mucus, bicarbonate, prostaglandins, antioxidants, and mucosal blood flow and offensive ones such as acid secretion, pepsin, and *Helicobacter pylori* (Longo, 2012 ▶). Annual frequency of the disease is globally about 8% and its outbreak rate is about 10% during the life (Sung et al., 2009 ▶). For many years, non-steroidal anti-inflammatory drugs (NSAIDs) have been implemented as the second risk factor in gastric ulcers etiology which can eventually result in bleeding and/or further gastrointestinal complications (Soll et al., 2012 ▶). By diminishing synthesis of prostaglandins, reducing blood flow in mucus and sub- mucosal layers of the alimentary system, and enhancing acid secretion and pepsin activity, NSAIDs cause such conditions in stomach which finally lead to formation of peptic ulcer (Longo, 2012 ▶). Human studies have shown that NSAIDs are responsible for more than 20% of peptic ulcers occurred in people receiving these drugs. So, in this study, we aimed to simulate this condition by using indomethacin to induce a model of gastric ulcer to evaluate better the possible protective effects of *Zataria multiflora* extract against NSAIDs-associated peptic ulcers (Yoshikawa et al., 1993). *Z. multiflora* is the flowered tip of Zataria genus which belongs to the Lamiaceae family. This plant grows in Iran, Pakistan and Afghanistan. In Iran, this plant is cultivated in Isfahan, Lorestan, Khouzestan and Fars provinces (Ghasemi-Dehkordi, 2002 ▶). This plant has been extensively used in Iranian traditional medicine for its antiseptic, pain relieving, anti-flatulence and intestine-soothing properties (Zomorodian et al., 2011). Total phenolic content of *Z. multifelora* extract was 283.43±11.06 mg/g gallic acid equivalent. (Sharafati-Chaleshtori et al., 2013 ▶). Phenol ingredients are mainly responsible for antibacterial properties of the extract and essential oil probably by causing multiple holes in bacteria cell membrane (Mashak and Moradi, 2012 ▶). In a study, this plant was shown to have antioxidant and protective activities against *Staphylococcus aureus* in meat (Sharafati- Chaleshtori et al., 2013 ▶). Linalool and p-cymene as two main constituents of this plant, are responsible for spasmolytic, antioxidant, anti-inflammatory and immunomodulatory properties of *Z. multiflora* (Khazdair et al., 2018 ▶). They were shown to inhibit acetylcholine and potassium chloride effects on ileum and uterus contractions, in rats (Gharib-Naseri et al., 2010 ▶). Also, carvacrol content of the extract can relax respiratory muscles by influencing histamine (H1), muscarinic and beta- adrenergic receptors (Boskabady et al., 2014 ▶). Moreover, *Z. multiflora* extract had comparable effects on white blood cells and blood level of interleukin 8 and malondialdehyde (MDA) in an animal model of chronic occlusive pulmonary disease (COPD) induced in guinea pigs, to those of dexamethasone (Boskabady and Gholami-Mohtaj, 2014). It also strengthens the mucus layer of duodenum and could protect against duodenal ulcer induced by cysteamine in rats (Minaiyan et al., 2005 ▶). Considering the beneficial properties of this plant, we tried to examine the anti-ulcerative effects of *Z. multiflora* hydroalcoholic extract (ZMHE) on gastric ulcer induced by indomethacin, in rats.

## Materials and Methods

**Chemicals**

*Z. multiflora* was purchased from local market in Isfahan. Indomethacin powder was purchased from Hakim pharmaceutical company (Tehran, Iran). Hemoglobin, pepsin, and orthodianizidine were purchased from Merck Co. (Darmstadt, Germany). Hematoxylin and Eosin (H&E) were procured from Padtan Teb Iran Company (Tehran, Iran). All other chemicals used were of analytical grade.

**Animals**

 In this study, 72 male Wistar rats (180±20 g) bred in animal house of Isfahan School of Pharmacy were used. During the experiment, rats were housed in separate groups (n=6) under standard conditions of temperature (22±2 C) and humidity (25±5%) with 12 hr/ 12 hr light/ darkness cycles. For 24 hr before the start of the experiment, animals were fasted while they had free access to tap water. All animal experiments were accomplished according to local Ethics guidelines approved by Isfahan University of Medical Sciences (approval No. IR.MUI.REC.1394.3.688).

**Preparation of hydro-alcoholic extract of** ***Z . multiflora***

The plant was authenticated in Pharmacognosy department of Isfahan School of Pharmacy and maceration method was used for full extraction. In this method, the powdered plant was soaked in ethanol and water (70:30) for two days and the procedure was repeated for three times to ensure full extraction (Minaiyan et al., 2011a). Then, the extract was condensed by rotary evaporator at 60 rpm at 45°C and dried by a freeze-drier (Minaiyan et al., 2006; Abed et al., 2012). Various suspensions at desired concentrations (20, 40 and 80 mg/ml) were prepared and the extract was analyzed by Folin- Ciocalteu method for polyphenol content which was expressed in terms of mg equal to gallic acid (Waterman and Mole., 1994).

**Induction of gastric ulcer**

Gastric ulcer was induced by injection of indomethacin (25 mg/kg, intraperitoneal (i.p.)) to animals that were already fasted for 24 hours (Borra et al., 2011). Oral and parenteral (i.p.) administration of the extract was done 2 and 1 hr before ulcer induction, respectively. Six hours later, the animals were sacrificed by inhalation of an overdose of diethyl ether. The abdomens were opened and the stomachs were excised while the both sides (cardiac and pyloric) were ligated appropriately. The contents of stomachs were removed, cut through their greater curvature and deposited on work sheets (Shahrani et al., 2007). Photographs were taken and tissues were evaluated for ulcer surface area and severity as mentioned below (Minaiyan et al., 2011b). The gastric tissue was kept at -18 C and -70 C for further histopathological examinations and myeloperoxidase (MPO) activity evaluations, respectively. 

**Animal groups**

Rats were randomly divided into 12 groups (6 rats in each) as follows:

Groups 1 and 2) Sham groups: orally and parenterally (i.p.) received vehicle (normal saline containing tween 20 (0.1%)) (5ml/kg) without prior gastric ulcer induction.

Groups 3 and 4) Control groups: received vehicle (5ml/kg; p.o. and i.p.) before gastric ulcer induction.

Groups 5-10) Extract groups: received ZMHE reconstituted in vehicle at selected doses (100, 200, 400 mg/kg; p.o. and i.p.) before gastric ulcer induction.

Groups 11 and 12) Reference groups: received ranitidine (25mg/kg; p.o. and i.p.) before gastric ulcer induction (Shahrani et al., 2007). All the oral and parenteral treatments were done 2 and 1 hr prior to ulcer induction, respectively. 

**Measurement of ulcer area**

Photographs were taken (Sony® camera, Japan), and analyzed by Fiji P image processing software Ver.32 (Fiji Contributors, Japan) for measuring the ulcer area and extent of ulcer. For evaluation of histopathological changes, gastric tissues of all animals were fixed in formalin 10%, sectioned into 4-6 µm slices, and stained with H&E. Then, the histopathological features such as inflammation, edema, bleeding and necrosis were evaluated by a pathologist who was blind to treatments. Insults were graded as follows: 1) normal mucosa; 2) hemorrhagic spots; 3) focal superficial mucosal cell loss; 4) focal superficial necrosis; 5) edema and 6) congestion (Parvan et al., 2017)

**Measurement of gastric pH and pepsin activity**

The gastric contents were collected and centrifuged at 2000 rpm for 10 min. Next, the supernatant was examined for pH by a digital pH meter (Shahrani et al., 2007).

Anson method was used to measure the pepsin activity of stomach contents. This method is based on the influence of stomach pepsin on hemoglobin, resulting in its decomposition and releasing amino acids; in this method, the absorbance is read by a UV spectrophotometer at 280 nm (Shahrani et al., 2007)

Briefly, 0.1 ml of gastric juice was diluted by 9.9 ml of normal saline and 0.5 ml of it was used as standard pepsin. To complete the reaction, trichloroacetic acid (TCA) solution was added and after 10 min, the contents of tubes were filtered and the absorbance was read by a calibrated spectrophotometer at 280 nm (Shahrani et al., 2007).

**Determination of MPO activity in gastric tissue**

To measure the MPO activity, 0.1 g of tissue was weighted and sliced. Then, the sample was homogenized in 1 ml of 50 M phosphate buffer at pH 6 containing 0.5% of Hexadecyl-trimethyl-ammonium bromide (HTAB) in an ice bath. The suspension was sonicated for 10 sec and then it was placed in a nitrogen tank to freeze but it was melted thereafter. This step was repeated for three times. The product was again sonicated and centrifuged at 4000 rpm for 15 min. Next, 0.1 ml of supernatant was collected and combined with 2.9 ml of phosphate buffer containing 0.005 mg/ml of hydrogen peroxide and 0.167 mg/ml of o-dianisidine hydrochloride. The absorbance was read at 250 nm from minute 0 to 3. The activity of MPO of gastric tissue was calculated in terms of unit / gram of tissue using the following equation (Motaghi et al., 2016):

MPO activity Ug=Aweight of the tissue    $A=\frac{10\times \mathrm{change in absorbance per minue}}{\mathrm{volume of supernatant}}$

**Statistical analysis**

Data was entered into Statistical Package for the Social Sciences (SPSS version 20.0) and presented as mean±standard deviation (SD) or median (range) for parametric or nonparametric values, respectively. For statistical evaluation, oneway analysis of variance (ANOVA) followed by Tukey’s *post hoc *test was used. For scoring values, Mann–Whitney Utest was used. In all analyzes, the significance level was set at p<0.05.

## Results

**Effect of ZMHE on gastric pH and pepsin activity**

ZMHE by oral route was effective to decrease the acid secretion in stomach and to elevate the pH value of its content and to decrease mean pepsin activity that was significant compared to control group at all applied doses (at least p<0.05) (Table 1). However, by injection route, this decrease in stomach acidity and pepsin activity, was just revealed a significant difference versus control group at the dose of 400 mg/kg (p<0.001).

**Effects of ZMHE on MPO activity in gastric tissue**

The results revealed that MPO activity declined in all extract and ranitidine-treated groups compared to control group (p<0.001) regardless of the route of ZMHE administration (Table 1). 

**Effects of ZMHE on macroscopic features in gastric ulcer**

ZMHE was effective following oral and parenteral administration in reducing ulcer area (cm^2^) as well as its severity (scores) compared to normal saline-treated controls (at least p<0.05). Overall, oral ZMHE was more effective than parenteral ZMHE (Table 2). Ranitidine either administered orally or by injection, was also effective to decrease the ulcer area and decreased the severity of gastric tissues insults (p<0.001) (Table 2).

**Table 1:** Effects of *Z. multiflora* hydroalcoholic extract (ZMHE) on different parameters of gastric ulcer induced by indomethacin, in rats.

**Group/Dose** **(mg/kg)**	**Route**	**pH**	**Pepsin activity** **(µg/15min)**	**MPO activity (U/g)**
**Sham**	p.o.	6.5±0.2	0.6±0.2	2.1± 0.3
**Control **	p.o	3.5±0.2	2.2±0.4	8.2±0.6
**ZMHE100**	p.o.	4.5±0.3^*^	1.2±0.3^**^	5.4±0.5 ^**^
**ZMHE200**	p.o.	5.4±0.2^***^	1.0±0.4 ^***^	3.3±0.4 ^***^
**ZMHE400**	p.o.	6.7±0.3 ^*^	0.8±0.3 ^***^	2.9±0.3 ^***^
**Ranitidine. 25**	p.o.	7.0±0.3	0.8±0.3 ^***^	2.7±0.2 ^***^
**Sham **	i.p.	6.5±0.3	0.7±0.3	2.3±0.3
**Control**	i.p.	3.3±0.2	2.5±0.5	9.1±0.8
**ZMHE100**	i.p.	3.5±0.2	2.2±0.5	5.5±0.5 ^***^
**ZMHE200**	i.p.	3.7±0.4	2.0±0.4	5.3±0.5 ^***^
**ZMHE400**	i.p.	5.7±0.3 ^***^	1.1±0.3 ^***^	4.1±0.3 ^***^
**Ranitidine.25**	i.p.	7.3±0.3 ^***^	1.3±0.4 ^**^	2.5±0.4 ^***^

Data are shown as mean±SD, (n=6). Sham and control groups received normal saline (5ml/kg). p.o.: oral and i.p.: intraperitoneal.

* p<0.05,

** p<0.01, and

*** p<0.001 show significant differences compared to related control group (ANOVA with Tukey's *post hoc* test)

**Effects of ZMHE on macroscopic features of gastric tissue**

**Figure 1:**
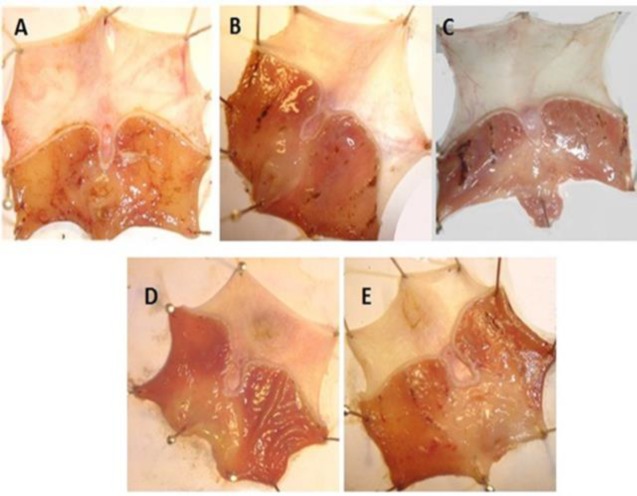
Macroscopic presentation of gastric tissue injuries induced by indomethacin, in rats.

In sham group, no ulcer and hemorrhagic spots were found (Figure 1A). In control group (peptic ulcer induced by indomethacin), several ulcer and hemorrhagic spots together with erythema, inflammation and edema especially in antrum region were evident (Figures 1B and C). In ranitidine-treated group, no ulcer was detected; however, some minor edema and inflammation were observed (Figure. 1D). In groups that received ZMHE, particularly at the dose of 400mg/kg, reduction in number of ulcer spots and attenuation in inflammation and ulcer severity as well as decreased edema and erythema were evident (Figure 1E).

**Table 2:** Effects of *Z. multiflora* hydroalcoholic extract (ZMHE) on ulcer markers of gastric ulcer induced by indomethacin, in rats.

**Group/Dose** **(mg/kg)**	**Route**	**Ulcer score (1-6)**	**Ulcer area (cm** ^2^ **)**
**Sham**	p.o.	1(1-1)	0.0±0.0
**Control **	p.o.	5(3-6)	1.9± 0.2
**ZMHE100**	p.o.	4(1-6)^*^	0.8± 0.2 ^***^
**ZMHE200**	p.o.	3.5(1-5) ^**^	0. 6±0.3 ^***^
**ZMHE400**	p.o.	2(1-3) ^***^	0.3±0.1 ^***^
**Ranitidine. 25**	p.o.	1.5(1-4) ^***^	0.3±0.2 ^***^
**Sham **	i.p.	1(1-1)	0
**Control**	i.p.	5(4-6)	1.8±0.2
**ZMHE100**	i.p.	4(1-6) ^*^	1.3±0.3 ^*^
**ZMHE200**	i.p.	3.5(1-6) ^**^	0.8± 0.3 ^***^
**ZMHE400**	i.p.	3(1-5) ^**^	0.5±0.3 ^***^
**Ranitidine. 25**	i.p.	1.5(1-3) ^***^	0.1±0.1 ^***^

Data are shown as median (range) for ulcer scores and mean±SD for ulcer area (n=6). Sham and control groups received normal saline (5 ml/kg). p.o.: oral and i.p.: intraperitoneal.

* p<0.05,

** p<0.01, and

*** p<0.001 indicate significant differences compared to related control group rats (Mann-Wheitney U test was used for ulcer score and ANOVA with Tukey's *post hoc* test, was used for ulcer area analysis)

A: normal; B and C: gastric ulcer in control group indicating hemorrhagic spots, superfacial erosions, edema and inflammation especially in antrum regions; D: gastric ulcer treated by ranitidine (25mg/kg); E: gastric ulcer treated by ZMHE (*Z. multiflora* hydroalcoholic extract, 400 mg/kg).

**Effects of ZMHE on histopathological features of gastric tissue**

**Figure 2:**
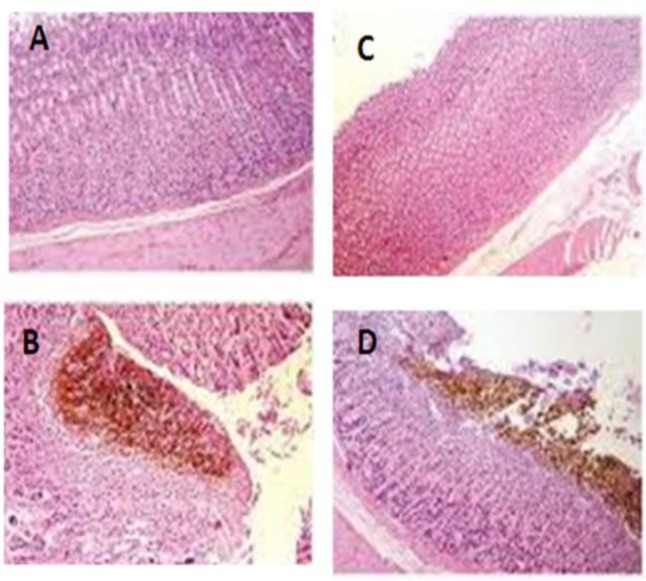
Microscopic evaluation of gastric tissue injuries induced by indomethacin in rats. A: normal tissue; B and D: gastric ulcer in control group; C: gastric ulcer treated by oral *Z. multiflora* hydroalcoholic extract at the dose of 400 mg/kg. H&E staining at X10 magnification.

Histopathological examination of stomach sections obtained from sham group showed normal mucosa and sub-mucosal layers as shown in Figure 2A. in control group, severe histopathological changes such as erythema, edema, inflammation and congestion were observed (Figures 2B and D).

In ZMHE groups (400 mg/kg, p.o.), mild inflammation and edema in mucosal layer were evident. Indeed, the results showed that necrosis and edema of mucosal layer meaningfully decreased by administration of ZMHE (Figure 2C). In ranitidine-treated group, nearly normal mucosal appearance and considerable reduction in ulcerative injuries, were seen. 

## Discussion

Indomethacin is an aril acetic acid derivative known as one of the most potent NSAIDs with many alimentary adverse effects (Furst et al., 2011 ▶) due to its inhibitory effect on prostaglandins synthesis. It can damage stomach tissue by increasing gastric acid and pepsin activity as well as enhancing lipid peroxidation and oxidative stress by producing free radicals in mucus (Suleyman et al., 2010 ▶; Hamauzu et al., 2008 ▶). Hence, we assessed the possible antiulcerative activity of *Z. multiflora* extract by measuring the gastric pH, pepsin and MPO activity in gastric tissue in addition to examination of macroscopic features and histopathological markers. In this study, ZMHE decreased the extent of ulcers compared to control group. Besides, it was revealed that ZMHE significantly reduced acid secretion. We know that pepsin activity is directly dependent on gastric pH and by diminishing acid secretion, pepsin activity will be proportionally decreased. This effect was found after administration of the three examined doses of ZMHE and the effect was dose-related. It means that local effect(s) of ZMHE in gastric tissue has an important role in this regard. Following parenteral administration, ZMHE had a significant effect only at the highest dose (i.e. 400mg/kg), suggesting a lesser potency compared to oral administration. It is assumed that quasi-tannin ingredients that abundantly exist in the extract can interact locally with pepsin and result in its inactivation. Moreover, due to high phenolic contents of ZMHE and their capacity to scavenge toxic radicals of oxygen, these ingredients may be responsible for its antioxidant property (Saei-Dehkordi et al., 2010 ▶) (Hamauzu et al., 2008 ▶). Removing oxygen free radicals is one of the probable mechanisms underlying anti-inflammatory, anti-ulcerative and ulcer-healing properties of *Z. multiflora* extract as diminished MPO activity, as an important inflammatory and oxidative biomarker, revealed this activity in extract-treated rats (Nakhaei et al., 2007 ▶, Samarghandian et al., 2016 ▶). Another effect that could be exerted by quasi-tannins of ZMHE is their local cytoprotective and mucus strengthening effects in gastric tissue which have been previously reported (Minaiyan, 2005 ▶). This might describe why did the oral intake of ZMHE was more effective than its administration via i.p. injection. 

It should be noted that flavonoids, essential oils and phenol ingredients of ZMHE are responsible for powerful antibacterial effects of this plant including its effect on *H. pylori* infection eradication (Hosseininejad, 2011 ▶, Sharafifar et al, 2007 ▶). *H. pylori* has an important role in gastric and duodenal ulcer induction and/or its aggravation in human subjects and antibacterial property of *Z. multiflora* can potentially be helpful in this regard (Sharififar et al., 2007 ▶). Albeit, more detailed studies are needed to validate these findings.

According to the results, it could be concluded that protective effect of *Z*. *multiflora* hydro-alcoholic extract against gastric ulcer caused by indomethacin, is mainly due to its counteracting effects against offensive factors like gastric acid secretion and pepsin activity or strengthening defensive factors such as increase in mucus and mucosal antioxidant capacity. Additionally, the results showed that oral administration of the extract had better anti-ulcer activity than the parenteral administration suggesting a more powerful local action for extract on alimentary tract.
